# Impact of Sub-MIC Eugenol on *Klebsiella pneumoniae* Biofilm Formation *via* Upregulation of *rcsB*

**DOI:** 10.3389/fvets.2022.945491

**Published:** 2022-07-12

**Authors:** Emad Mohammed Elken, Zi-ning Tan, Qian Wang, Xiu-yun Jiang, Yu Wang, Yi-ming Wang, Hong-xia Ma

**Affiliations:** ^1^College of Animal Medicine, Jilin Agricultural University, Changchun, China; ^2^The Key Laboratory of New Veterinary Drug Research and Development of Jilin Province, Jilin Agricultural University, Changchun, China; ^3^Animal Production Department, Faculty of Agriculture, Al-Azhar University, Nasr City, Egypt; ^4^The 3nd Affiliated Clinical Hospital of Changchun University of Chinese Medicine, Changchun, China; ^5^College of Life Science, Jilin Agricultural University, Changchun, China; ^6^The Engineering Research Center of Bioreactor and Drug Development, Ministry of Education, Jilin Agricultural University, Changchun, China

**Keywords:** eugenol, sub-MIC, biofilm formation, *Klebsiella pneumoniae*, rcsB

## Abstract

The Rcs phosphorelay system is present in many members of the *Enterobacteriaceae*. The aim of this study was to illustrate the possible mechanisms of eugenol on ultimate targets of *Klebsiella pneumoniae* (*K. pneumoniae*) Rcs phosphorelay, *rcsB*, and impact on biofilm formation. The minimum inhibitory concentration (MIC) of eugenol against *K. pneumoniae* KP1 and KP1 Δ*rcsB* strain was determined using the 2-fold micro-dilution method. Biofilm was measured by crystal violet staining. Transcriptome sequencing was performed to investigate sub-MIC eugenol on *K. pneumoniae*, and gene expression at mRNA level was analyzed by RT-qPCR. *In vitro* biofilm formation test and molecular docking were used to evaluate the effect of eugenol and to predict potential interactions with RcsB. MicroScale Thermophoresis (MST) was conducted for further validation. MIC of eugenol against *K. pneumoniae* KP1 and KP1 Δ*rcsB* strain was both 200 μg/ml. Transcriptome sequencing and RT-qPCR results indicated that *rpmg, degP, rnpA*, and *dapD* were downregulated, while *rcsB, rcsD, rcsA, yiaG*, and *yiaD* were upregulated in the eugenol-treated group. Δ*rcsB* exhibited a weakened biofilm formation capacity. Additional isopropyl-β-d-thiogalactoside (IPTG) hinders biofilm formation, while sub-MIC eugenol could promote biofilm formation greatly. Docking analysis revealed that eugenol forms more hydrophobic bonds than hydrogen bonds. MST assay also showed a weak binding affinity between eugenol and RcsB. These results provide significant evidence that *rcsB* plays a key role in *K. pneumoniae* biofilm formation. Sub-MIC eugenol facilitates biofilm formation to a large extent instead of inhibiting it. Our findings reveal the potential risk of natural anti-biofilm ingredients at sub-MIC to treat drug-resistance bacteria.

## Introduction

Antimicrobial resistance (AMR) has increased the challenge in today's livestock and human health globally. *Klebsiella pneumoniae* is one of the opportunistic pathogens that cause numerous diseases, such as bacteremia, pneumonia, septicemia, respiratory and urinary tract infections, mastitis, pyometra, and enteritis in cattle, goats, and companion animals (1). Globally, an increase in the incidence of hypervirulent and multidrug-resistant *K. pneumoniae* is considered a consequence of currently excessive use of antibiotics (2, 3). Although *K. pneumoniae* is believed to be less risky for animal production for a long time, its prevention and control have been necessitated due to the emergence of hypervirulent and multidrug-resistant strains of *K. pneumoniae* (4). For instance, it has also been noticed that loss of milk production as a result of mastitis and deaths of livestock is due to these multidrug-resistant strains of *K. pneumoniae* (5).

Biofilm resistance ability is one of the major barriers to the treatment of infectious diseases, as it prevents the antibiotics from interring the bacterial cells. The formation of bacterial biofilm is a dynamic process that depends on bacterial structure, function, and composition (6, 7). Bacterial extracellular polysaccharides (EPSs) play a major role in stabilizing the structure and function of bacterial biofilms (8).

The Regulator of Capsule Synthesis (Rcs) phosphorelay system was originally identified during a screening study on *Escherichia coli* for necessary genes responsible for capsular polysaccharide (CPS) synthesis (9). The Rcs phosphorelay system is composed of core proteins RcsB, RcsC, and RcsD (10). The phosphoryl group is transferred to a phosphotransfer protein RcsD (known as membrane-spanning protein) by RcsC and finally to RcsB (11). The previous study has proved that in the absence of external phosphatases, the Rcs activation signal passed to RcsB can be long-lived. Despite this, RcsB can have significant constitutive activity in the absence of RcsC or RcsD phosphorelay but through other pathways to carry out regulation. In addition, several auxiliary regulatory proteins such as RcsA, GadE, BglJ, MatA (EcpR), TviA, DctR, RflM, and RmpA act independently for phosphorylation (12, 13). The biofilm formation and regulation of CPS are majorly dependent on the Rcs phosphorelay (14, 15).

Eugenol is a phenolic component occurring in many aromatic plants of essential oil and other sources such as cinnamon extract, clove bud oil, and many others. Many studies have demonstrated antimicrobial, antioxidant, anti-inflammatory, antifungal, antiviral, anticancer, analgesic, and antispasmodic activities for eugenol (16). The double bond located at the γ position of the methyl group and α and β positions of the side chain is responsible for eugenol-mediated antimicrobial activity (17).

The application of eugenol can prevent the growth of pathogenic biofilm formed by *K. pneumoniae* (18). With this background, this study aimed to investigate the impact of sub-minimum inhibitory concentration (MIC) eugenol on *K. pneumoniae* biofilm formation and possible interactions with RcsB.

## Methods

### Chemicals, Bacteria, and Culture Medium

Eugenol (purity ≥98.5%) was purchased from Shanghai Yuan Ye Bio-Technology Co., Ltd. Clinical *K. pneumonia* isolate KP1 and KP1Δ*rcsB* strain were preserved in our laboratory. Luria-Bertani (LB) medium was obtained from Qingdao Hope Bio-Technology Co., Ltd (Qingdao, China).

### Determination of MIC

The MIC of eugenol against *K. pneumonia* wild-type KP1 and Δ*rcsB* strain was determined using the broth micro-dilution method. Bacterial suspension at 10^6^ CFU/ml was used for serial dilution and inoculated to an equal volume of eugenol. The negative and positive control tubes were set as LB broth only and LB with bacteria, respectively. The value of the lowest concentration that inhibits the growth of bacteria after incubation at 37°C for 12 h was termed MIC. This assay was performed in triplicate at separate times.

### Detection of Biofilm Formation

The biofilm formation of KP1 and Δ*rcsB* strain was determined according to a previous study with slight modifications (19). In brief, the bacteria grown in LB broth were cultured to an OD_600_ of 0.5 (10^8^ CFU/ml). A 96-well microtiter plate was used for equal distribution of the suspension. The tubes containing LB medium only were set as the negative control. The plates were incubated at 37°C for 24 h to facilitate biofilm formation in a static culture. Contents were removed gently after incubation with tap water. Notably, 200 μl of phosphate buffer saline (PBS, pH 7.2) was used for wells washing four times to remove any unbound cells. To stain the remaining adherent bacteria, 50 μl of crystal violet (1%) was added to the wells followed by keeping them at room temperature for 15 min. Distilled water was used for the removal of the excess stain, and the pallet was subjected to drying. The stained biofilm was solubilized with 95% ethanol, and the total biofilm mass was quantified by measuring the OD_570_ using the Universal Microplate Reader (Thermo Fisher Scientific). Samples were assayed in 6 replicates and performed thrice. The previous study was adopted for interpretation of the biofilm production (Table 1) (20).

**Table 1:** Interpretation of biofilm production.

**Biofilm production**	**Average OD value**
Non	≤ODc
Weak	ODc < OD≤2 ODc
Moderate	2 ODc < OD≤4ODc
Strong	>4 ODc

*ODc, average OD value of negative control well + 3 × standard deviation of OD value of negative control well*.

### Treatment of Samples, RNA Extraction, Library Construction, and Transcriptome Sequencing

Transcriptome sequencing was described in a formerly published paper in our laboratory. In brief, *K. pneumoniae* was cultured to reach 0.5 at OD_600_. Then, eugenol was added to a final concentration of 1/4 MIC and further cultured for 1 h. In control, LB medium was added instead of eugenol. The total RNA was extracted and digested with DNase I in compliance with the manufacturer's instructions. The quality and quantity of RNA were measured using the Thermo Scientific NanoDrop 2000 UV-Vis Spectrophotometer. Complementary probe sequences were applied for the elimination of the prokaryotic rRNAs. The template for the synthesis of the first strand of cDNA was obtained by random fragmentation of the depleted rRNA into small pieces using a fragmentation buffer. Subsequently, a buffer, dNTPs, dATP, dGTP, dCUP, RNaseH, and DNA polymerase I were added for the synthesis of the second strand of cDNA. The samples were sent to Biomarker Technology for library construction and sequencing using Illumina HiSeq 2500 platform. Genome-based reads mapping reference was adopted for transcriptome analysis. High-quality reads were mapped with reference *K. pneumoniae* strain KP52.145 sequence (GenBank: FO834906.1) to get position and characteristic information.

### RT-qPCR Analysis

The expression of *rpmG, degP, rnpA, dapD, rcsB, rcsD, rcsA, yiaG*, and *yiaD* genes at the mRNA level was selected from transcriptome sequencing and evaluated *via* RT-qPCR. Bacteria were prepared as mentioned in the “Transcriptome Sequencing” section. In the control group, LB broth was added instead of eugenol. The total RNA was extracted using the method as previously described and was then reversely transcribed to cDNA. Then, cDNA was used as a template for RT-qPCR amplification in the same reaction tube using PerfectStartTM Green RT-qPCR SuperMix. RT-qPCR was carried out in an Applied Biosystems 7500 RT-qPCR System (USA). The sequences of RT-qPCR primers used are shown in Table 2. The amplifications were performed in 20 μl reaction mixtures containing 10 μl PerfectStartTM Green RT-qPCR SuperMix, 0.5 μl forward and reverse primer each, 8.0 μl nuclease-free water, and 1.0 μl template, respectively. 16SrRNA of the strain was used as a reference gene. The reaction condition was set as a two-step method as follows: one cycle at 94°C for 30 s, then 40 cycles consisting of denaturation were at 95°C for 5 s, and signal collection at 60°C for 34 s. All templates were run in triplicates.

**Table 2:** Sequences of PCR and RT-qPCR primers used in this study.

**Primer name**	**Sequence (5^′^-3^′^)**	**Product size (bp)**
*rcsB*	F: CCTCGAGATGAACACTATGAACG	651
	R: GGAATTCTTACTCTTTGTCCGTC	
*rpmG*	F: CGCTGGTACTGGTCACTTCTACAC	97
	R: GTGCTGACGTACAACCGGATCG	
*degP*	F: GGCACCGAACTGAACTCTGAGC	86
	R: GAACCTGGCATTACCTGGCTGAC	
*rnpA*	F: GTCTTCCAGCAGCCACAACGG	148
	R: GCGTCAGACGTTTAATCCGATTGC	
*dapD*	F: CACTGTGCTGATGCCGTCCTAC	147
	R: TCCAGTACGCCACCGATGCC	
*rcsB*	F: ACCATCAGCAGCCAGAAGAAATCG	89
	R: AGCGAGACGGAAGAGAGGTAGTTC	
*rcsD*	F: GCCACCGACATCAGCACCAC	101
	R: AGCAGCAGCGGCAGAAGAATG	
*rcsA*	F: ATTTGTGCAGCTATACCCGGTTGG	97
	R: AGATCCGCAGCATTGTTGACCTC	
*yiaG*	F: AAGATCCCGTGTTTGAACTGCTG	94
	R: AACACAGTGGACTTACGGTTTGAGG	
*yiaD*	F: GGTTACTACATGGACGTGCAGGAAG	115
	R: AGGTGACGTTATTCGGCATGTTCAG	
16SrRNA	F: TGTCGTCAGCTCGTGTTGTG	130
	R: ATCCCCACCTTCCTCCAGTT	

### Inhibitory Effect of Eugenol on Bacterial Biofilm Formation

The biofilm formation of different groups was as follows: plasmid pET-28a-*rcsB* transferred to Δ*rcsB* competent cell was set as Δ*rcsB* + RcsB group, plasmid pET-28a-*rcsB* transferred to KP1 competent cell was set as KP1 + RcsB group, moreover, Δ*rcsB* + RcsB + Eu, Δ*rcsB* + RcsB + isopropyl-β-d-thiogalactoside (IPTG), Δ*rcsB* + RcsB + IPTG + Eu, KP1 + RcsB + Eu, KP1 + RcsB + IPTG, and KP1 + RcsB + IPTG + Eu were also set, respectively. In Δ*rcsB* + RcsB + IPTG and KP1 + RcsB + IPTG groups, 0.1 mmol/L IPTG was first added and cultured for 4 h to contribute to the induction and then cultured for 24 h. For Δ*rcsB* + RcsB + IPTG + Eu and KP1 + RcsB + IPTG + Eu groups, in addition to the above treatment, eugenol was cocultured with the mixture with a final concentration of 1/4 MIC at 37°C for 24 h. The biofilm formation was determined with the same method as mentioned in the sections above. The negative and positive controls were set on the basis of tubes containing LB only and LB with Δ*rcsB* or KP1, respectively.

### Molecular Docking

To gain further insight into the interaction between eugenol and RcsB, molecular docking was performed. ChemBioDraw Ultra 17.0 was used to draw the structure of eugenol. The three-dimensional structure of RcsB (PDB ID: 2kx7) was selected and downloaded from the Protein Data Bank. RcsB and eugenol were converted into PDBQT format using AutodockTools 1.5.6. Autodock Vina 1.1.2 was adopted for molecular docking research. To increase the accuracy of calculation, the parameter exhaustiveness was set to 20. Unless otherwise specified, all other parameters adopted default values. Finally, the conformation with the highest score was selected to analyze the results with the Discovery Studio 2019 client.

### MicroScale Thermophoresis Analysis

To determine the equilibration dissociation constant (KD) of eugenol to RcsB, MicroScale Thermophoresis (MST) measurements were performed by Protein Preparation and Identification Platform, Protein Research Technology Center, Tsinghua University, using a Monolith NT.115 device (201610-BR-N007, Nanotemper Technologies, Munich, Germany). RcsB, with a calculated MW of 27.5 kDa, was already expressed, purified, and stored in the laboratory. Eugenol was added to samples with a final concentration of 50 μg/ml containing increasing concentrations of RcsB (0–100 nM). Then, a 1:1 serial dilution of protein and eugenol stock solution was prepared in PBS containing 0.05% Tween-20. The samples were then transferred into specialized glass capillaries (Monolith NT.155 premium capillaries MO-K022; Nanotemper Technologies, Munich, Germany). For all experiments, standard parameters were used as recommended by the manufacturer. The data were evaluated through the thermophoresis effect using the manufacturer-supplied NT analysis software.

### Statistical Analysis

All data were expressed as mean ± standard deviation. Statistical analysis was tested using Graphpad Prism 8 using Student's *t*-test, and *P* < 0.05, *P* < 0.01, and *P* < 0.001 were considered statistically significant and marked as ^“*,”^^“**,”^ and ^“***,”^ respectively, in all comparisons.

## Results

### Minimum Inhibitory Concentration

As shown in Table 3, the MIC value of eugenol was 200 μg/ml against both KP1 and Δ*rcsB* strains.

**Table 3:** Minimum inhibitory concentration (MIC) of eugenol against tested bacteria.

**Bacteria**	**Eugenol**
	**MIC (μg/mL)**
*Klebsiella pneumoniae* KP1	200
**Δ** *rcsB*	200

### Biofilm Formation

**Figure 1:**
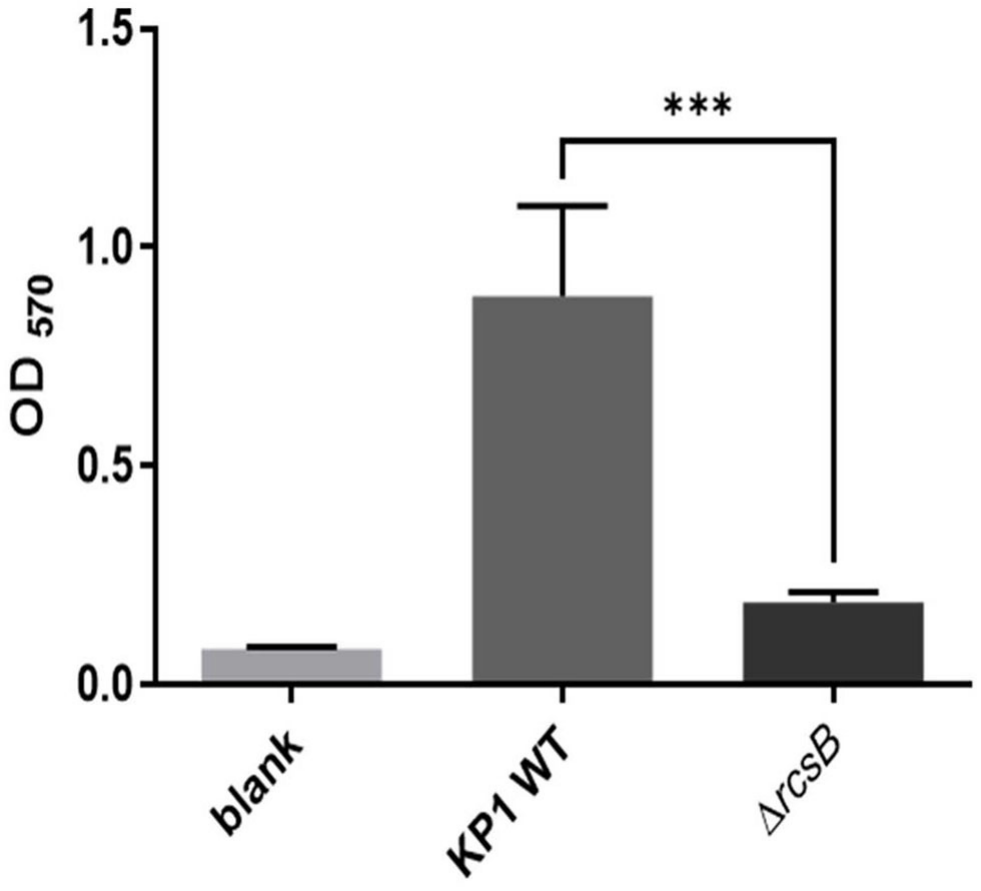
Biofilm formation of *Klebsiella pneumonia* KP1, Δ*rcsB*, and sterile LB broth as blank. The *** denotes *P* < 0.001.

Crystal violet assessment confirmed that wild-type KP1 grown on LB medium at 37°C for 24 h resulted in a strong biofilm formation. However, Δ*rcsB* strain almost loses the biofilm formation capacity under similar conditions (Figure 1).

### Transcriptome Sequencing

The data were available in the NCBI SRA database (SRA accession: PRJNA504310). Among these, 5,779 reference genes were detected including 4,514 known genes in the control group and 4,561 genes in the eugenol treated group. Based on the false discovery rate (FDR) threshold, 890 significant DEGs were identified where 771 genes were upregulated and the rest 119 genes were downregulated between the two groups.

### Expression Level of Selected Genes

**Figure 2:**
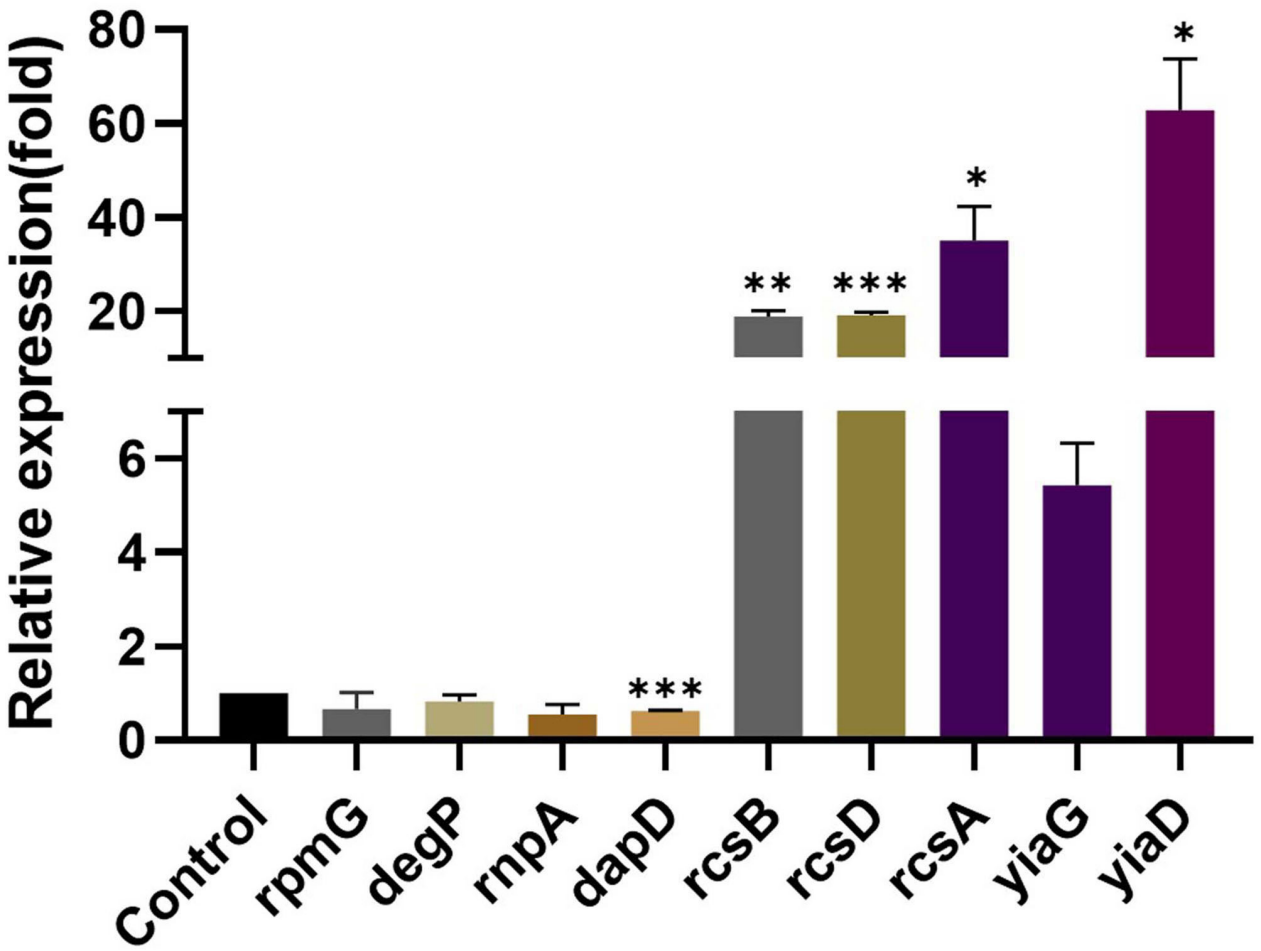
Relative expression of *rpmG, degP, rnpA, dapD, rcsB, rcsD, rcsA, yiaG*, and *yiaD*. The *, **, and *** denotes *P* < 0.05, *P* < 0.01, and *P* < 0.001 respectively.

The results of RT-qPCR showed that the relative expression of 50S ribosomal protein gene *rpmG*, serine endoprotease gene *degP*, and ribonuclease P protein component *rnpA* in the KP wild type was downregulated after eugenol treatment. No significant difference was observed compared with the nontreated group. As shown in Figure 2, the expression of 2,3,4,5-tetrahydropyridine-2,6-carboxylate *N*-succinyltransferase synthesis gene *dapD* was significantly lower than the control group. Oppositely, rcs phosphorelay system core genes *rcsB, rcsD*, and *rcsA* and transcriptional regulator gene *yiaG* showed a significant upregulation trend after eugenol treatment. Expression of OmpA family lipoprotein gene *yiaD* was also higher than control. The expression profile of all tested genes was in agreement with the FPKM dataset of transcriptome sequencing except for *degP*.

### Effect of Eugenol on Biofilm Formation

**Figure 3:**
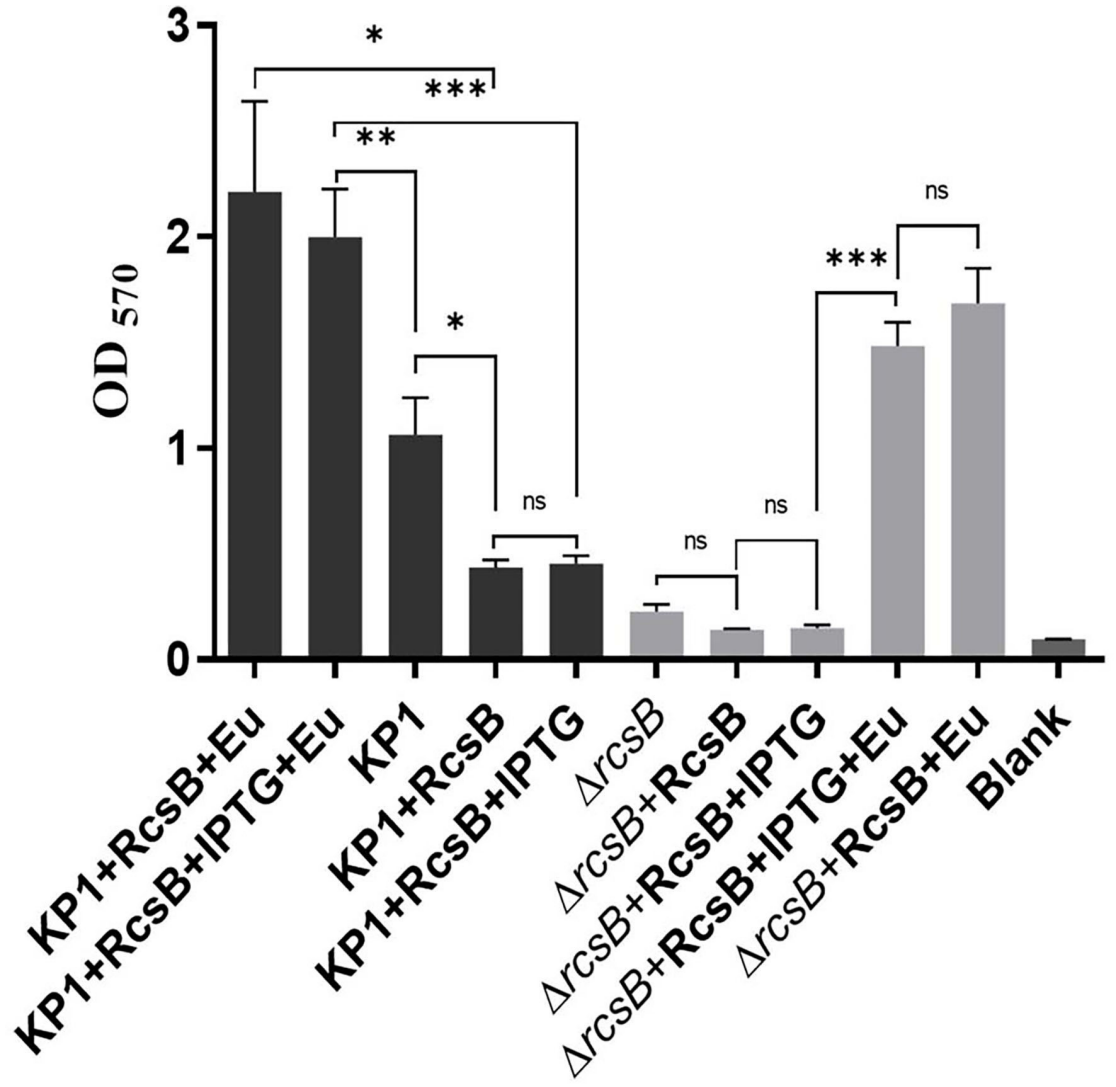
Effect of eugenol on biofilm formation. The *, **, and *** denotes *P* < 0.05, *P* < 0.01, and *P* < 0.001 respectively.

It can be seen from Figure 3 that plasmid pET-28a-*rcsB* transformation can hardly recover biofilm formation of Δ*rcsB* competent cells compared with Δ*rcsB* only. Besides, a decrease of biofilm in Δ*rcsB* + RcsB + IPTG, Δ*rcsB* + RcsB + Eu, Δ*rcsB* + RcsB + IPTG + Eu, KP1 + RcsB + Eu, and KP1 + RcsB + IPTG + Eu groups was observed after IPTG induction. However, nonsignificance was found within Δ*rcsB* + RcsB + IPTG, Δ*rcsB* + RcsB, KP1 + RcsB, and KP1 + RcsB + IPTG groups.

Oppositely, all groups after adding sub-MIC eugenol exhibited an obvious biofilm increase trend compared with nontreatment groups, which reflects a strong biofilm promoting effect of eugenol. Biofilm in both KP1 + RcsB + IPTG + Eu and Δ*rcsB* + RcsB + IPTG + Eu groups was significantly higher than that in the noneugenol group. This result was consistent with transcriptome sequencing and RT-qPCR. Moreover, a significant difference (*P* = 0.0075) was observed between biofilm formation of KP1 + RcsB + IPTG + Eu and KP1 regardless of IPTG inhibition. Likewise, the same trend was also shown in the KP1 + RcsB + Eu group compared with the KP1 group. Based on the above results, we found that sub-MIC eugenol contributes greatly during the biofilm formation process alone or by induction of *rcsB*.

### Analysis of Molecular Docking

**Figure 4:**
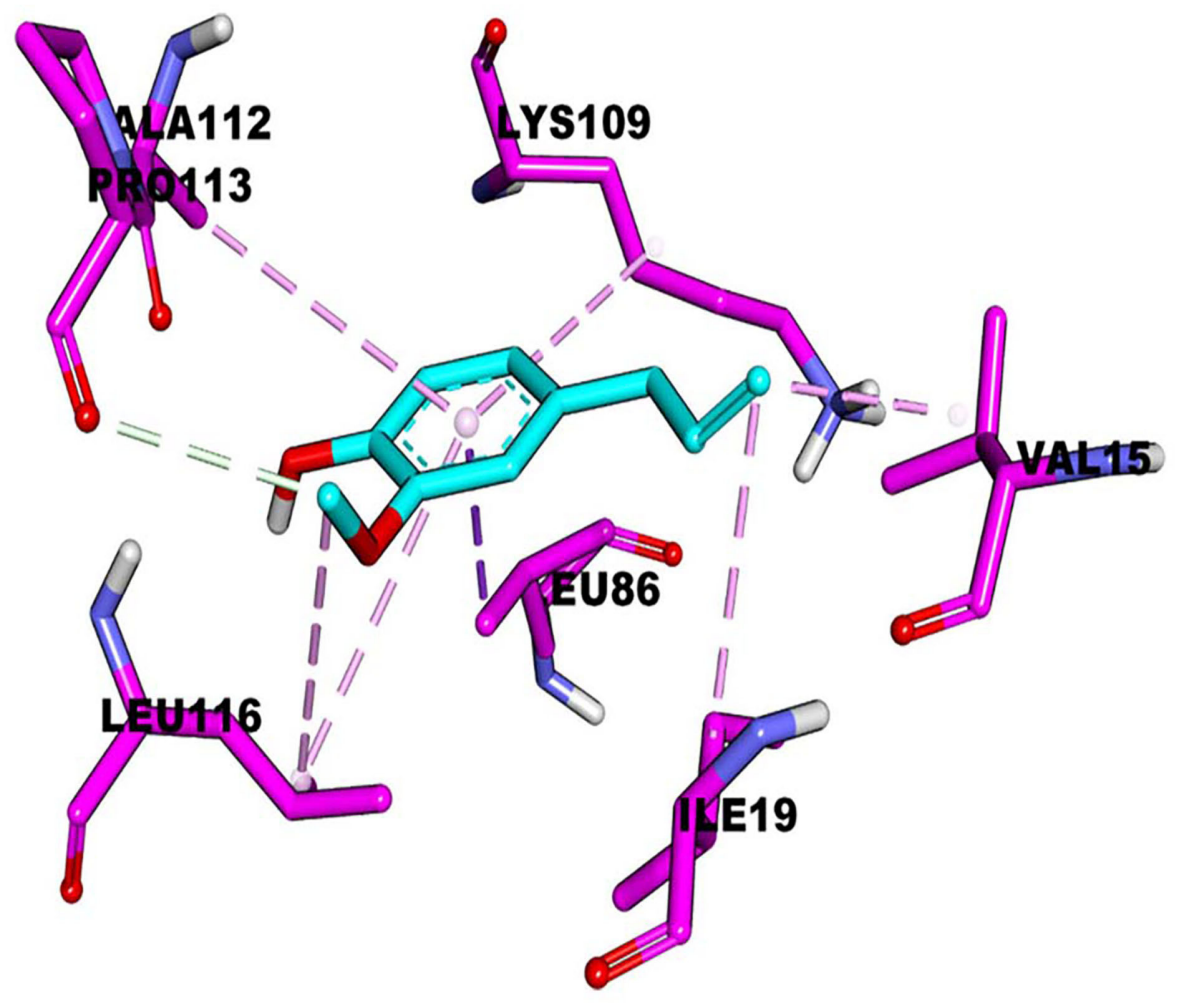
Molecular docking of eugenol with RcsB.

The active pockets of protein RcsB were docked by eugenol to clarify their mode of action at the molecular level. The affinity was −4.3 kcal/mol. The theoretical combination mode is shown in Figure 4. It can be seen from the surface map that small molecules and protein surfaces formed more bonds, which provides the possibility for small molecules to bind here. However, there are six hydrophobic bonds and only one hydrogen bond out of total. The interactions are shown in Table 4. All these interactions make the RcsB and eugenol pound Lig form a complex.

**Table 4:** Binding affinity of RcsB with eugenol *via* molecular docking.

**Bond-forming amino acid**	**Distance**	**Bonding type**	
Lig:C11–proteinB:PRO113:O	3.48293	Hydrogen bond	Carbon hydrogen bond
ProteinB:LEU86:BD1–Lig	3.63002	Hydrophobic	Pi-Sigma
Lig:C9–proteinB:VAL15	3.93742	Hydrophobic	Alkyl
Lig:C9–proteinB:ILE19	5.16117	Hydrophobic	Alkyl
Lig–proteinB:LYS109	5.27615	Hydrophobic	Pi-Alkyl
Lig–proteinB:ALA112	5.3649	Hydrophobic	Pi-Alkyl
Lig–proteinB:LEU116	5.09644	Hydrophobic	Pi-Alkyl

### Binding Affinity Analysis of Eugenol to RcsB

**Figure 5:**
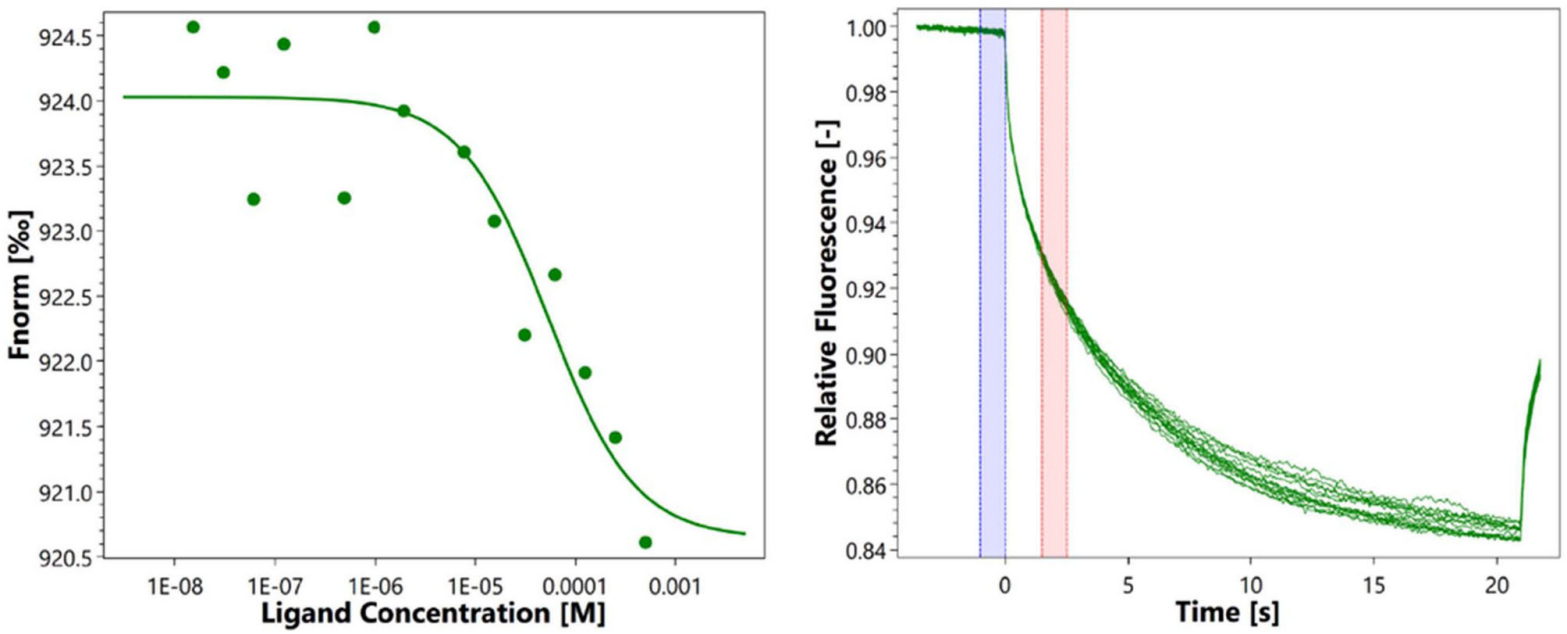
Microscale thermophoresis measurements of eugenol to RcsB.

Microscale thermophoresis measurements were used to determine the equilibration dissociation constant (KD) of eugenol to RcsB. The signal-to-noise ratio of RcsB was 7.3. The RcsB exhibits a binding affinity to eugenol with a resulting KD of 53.4 μM at a target concentration of 100 nM (Figure 5). The result of MST further proved the slight interaction of eugenol with RcsB.

## Discussion

Previous investigation revealed that the production of cell surface structures was dependent on the Rcs cascade complex signal transduction system (11), and peptidoglycan synthesis was responsible for bacteria growth (21). Several virulence factors such as CPS, lipopolysaccharide (LPS), and biofilm formation-related factors responsible for pathogenicity were identified in *K. pneumoniae* (22). Biofilm is also considered an important factor of virulence in pathogenic bacteria. The general architecture including depth and 3D structure is determined by exopolymeric matrix component colonic acid (23). Moreover, the expression of extracellular polysaccharide colanic acid is decreased by the deactivation of the Rcs phosphorelay (9).

Biofilm is often described as a shelter that protects bacteria from hostile surroundings as well as one of the strategic ways to develop resistance against antimicrobials in which those formed biofilms are more resistant than their planktonic counterparts up to 1,000 times. It also offers a reservoir for chronic infection. However, the adjustment of biofilm to heterogeneous physicochemical conditions is necessary due to its exposure to several stresses. For instance, modifications of the LPS in Gram-negative bacteria could be caused by such adaptations. It is considered a highly dynamic structure of the bacterial outer membrane that responds to environmental changes in the surrounding (24).

The Rcs phosphorelay, presented in many *Enterobacteriaceae* genera, can regulate the expression of important genes in large numbers for cell division and stationary-phase sigma factor activity, maintaining the integrity of cell wall, motility, virulence, and biofilm development (11). In this study, a vital role of RcsB was noted for *K. pneumoniae* biofilm formation, while low production of biofilm was observed in Δ*rcsB* in comparison to the wild-type strain. A similar effect on the biofilm formation and growth of *Edwardsiella tarda* was reported by Xu et al. (25). Another report mentioned that the *rcsD* mutant of *P. mirabilis* was deficient in biofilm formation, where the contribution of Rcs phosphorelay to biofilm formation was also validated (26). Reduced biofilm production of Δ*rcsB* mutant is due to its fragile architecture. An easy detachment of biofilm from the surface is due to complex architecture and significant depth (25). In another study on *E. coli*, inactivation of the Rcs system was found that can lead to a decrement in extracellular polysaccharide colonic acid expression, which participates in overall biofilm architecture (9, 23).

In this study, lesser production of biofilm was observed. Transformation of plasmid pET-28a-*rcsB* did not enhance biofilm formation in either group. It is evident from the results that biofilm formation was directly affected by the expression of *rcsB*. However, a sharp decrease was found in KP1 competent cells transferred with plasmid pET-28a-*rcsB*. Although Δ*rcsB* had a weakened biofilm formation capacity, the biofilm of both Δ*rcsB* and KP1 competent cells was weakened than the original strains, indicating a competent state detrimental to bacteria. Beyond our expectation, instead of facilitating biofilm formation, IPTG induction resulted in biofilm loss during 4 h incubation. The trend was reflected by Δ*rcsB* + RcsB + IPTG, Δ*rcsB* + RcsB + IPTG + Eu, KP1 + RcsB + Eu, and KP1 + RcsB + IPTG + Eu groups. Among them, the biofilm of Δ*rcsB* + RcsB + IPTG was significantly lower than Δ*rcsB* + RcsB + IPTG + Eu. Inversely, no significant difference was found between KP1 + RcsB and KP1 + RcsB + IPTG, as well as Δ*rcsB* + RcsB and Δ*rcsB* + RcsB + IPTG groups. We speculate that induction by IPTG may create a favorable environment and let bacteria release “fake” signals to transcriptional regulatory factors to slow down the overall expression of genes essential for CPS synthesis and thus lower the biofilm level. This effect may suppress the single upregulation and a series of hereafter biofilm-related gene regulations. The differences between Δ*rcsB* and KP1 competent cells could be attributed to the *rcsB* gene existing in the KP1 chromosome, which causes the sensitivity difference between the two strains to ambient pressure. The exact mechanism needs to be further studied. Interestingly, regardless of IPTG's inhibitory effect, all groups were found to exhibit an obvious biofilm increase, especially in KP1 + RcsB + IPTG + Eu and Δ*rcsB* + RcsB + IPTG + Eu groups after adding sub-MIC eugenol compared with nontreatment groups, which reflect a strong biofilm promoting effect of eugenol. Through transcriptome sequencing and RT-qPCR, upregulations of mRNA expression of Rcs phosphorelay system response regulator *rcsB*, histidine-containing phosphotransfer domain *rcsD*, unstable substrate of the Lon protease *rcsA*, as well as a transcriptional regulator gene *yiaG* were analyzed and validated, which was in agreement with the *in vitro* biofilm formation result. Besides, molecular docking can reproduce the strength between the 3-D dimensional structure of the receptor and the ligand. An energy-binding manner between the ligand and the active site of the receptor can be evaluated. The molecular docking studies give a reasonable explanation for the interaction between RcsB and eugenol. Through analysis, eugenol could form more hydrophobic bonds that will weaken stronger bond such as hydrogen bond with RcsB. Concerning the difference, we think eugenol can target RcsB but bind normally. To better understand the binding affinity between RcsB and eugenol, MST analysis was performed. Eugenol was further proved that it can interact with RcsB.

Based on the results obtained, we found that sub-MIC eugenol contributes greatly during the biofilm formation process alone or by induction of *rcsB* expression, which was converse to its biological property at MIC. Many previous studies reported that eugenol significantly reduced or eradicated biofilms formed by *Streptococci* (27), *Candida dubliniensis* (28), *Staphylococcus aureus* (18), *E. coli* O157:H7 (29), *Porphyromonas gingivalis* (30), and *Listeria monocytogenes* (31).

Inhibition of biofilm formation without affecting bacterial activity at an early stage in Streptococcus mutants was reported previously (32). The absence of *rcsB* in Salmonella weakens biofilm formation phenotypes; besides, the absence of the phosphorelay pathway of other components such as RcsD, RcsC, and RcsA conforms to normal biofilm phenotypes (33). However, the mechanism and impact of sub-MIC eugenol against various pathogens have not yet been fully understood. Our results showed that sub-MIC natural products not only can hardly reduce or eradicate bacteria within or at the biofilm stage effectively but also will facilitate the formation at an early stage. This phenomenon offers an explanation of induced drug resistance to some extent. Therefore, more attention should be paid to the potential risk of low-dose chemical ingredient administration. The vital role of two component systems and contributions to drug resistance development and bacterial pathogenicity should not be ignored.

## Conclusion

*rcsB* is critical and plays a positive role in *K. pneumonia* biofilm formation. Sub-MIC eugenol exerted a biofilm-promoting property *via* upregulating Rcs-related genes. Our study provides a new perspective and evidence for natural antibacterial ingredient research and the potential risk of sub-MIC natural ingredients to treat drug-resistance bacteria.

## Data Availability Statement

The datasets presented in this study can be found in online repositories. The names of the repository/repositories and accession number(s) can be found in the article/Supplementary Material.

## Author Contributions

EE and Z-nT performed the experiment. EE, YW, and Y-mW contributed to drafting and revising the manuscript. QW, X-yJ, and Y-mW participated in the design of the study and statistical analysis. H-xM provided constructive advice and checked the final manuscript. All authors have read and approved the final manuscript.

## Funding

This study was supported by the Science and Technology Project of Jilin Provincial Department of Education 13th Five-Year Plan (Grant number: JJKH20200359KJ), Introduction of Excellent Doctor Protocol of Jilin Agricultural University.

## Conflict of Interest

The authors declare that the research was conducted in the absence of any commercial or financial relationships that could be construed as a potential conflict of interest.

## Publisher's Note

All claims expressed in this article are solely those of the authors and do not necessarily represent those of their affiliated organizations, or those of the publisher, the editors and the reviewers. Any product that may be evaluated in this article, or claim that may be made by its manufacturer, is not guaranteed or endorsed by the publisher.

## Abbreviations

AMR: antimicrobial resistance
*K. pneumoniae*: *Klebsiella pneumoniae*
MIC: minimum inhibitory concentration
MST: MicroScale Thermophoresis
Rcs: regulator of capsule synthesis
EPS: extracellular polysaccharides
IPTG: isopropyl-β-d-thiogalactoside
CPS: capsular polysaccharide
LB: Luria-Bertani
FDR: false discovery rates
DEG: differential expression gene
LPS: lipopolysaccharide.
