# Supplementary figures and images for: Impact of Sub-MIC Eugenol on Klebsiella pneumoniae Biofilm Formation via Upregulation of rcsB
Source: Front Vet Sci. 2022 Jul 12;9:945491. doi: 10.3389/fvets.2022.945491 (PMC9315372; doi:10.3389/fvets.2022.945491)

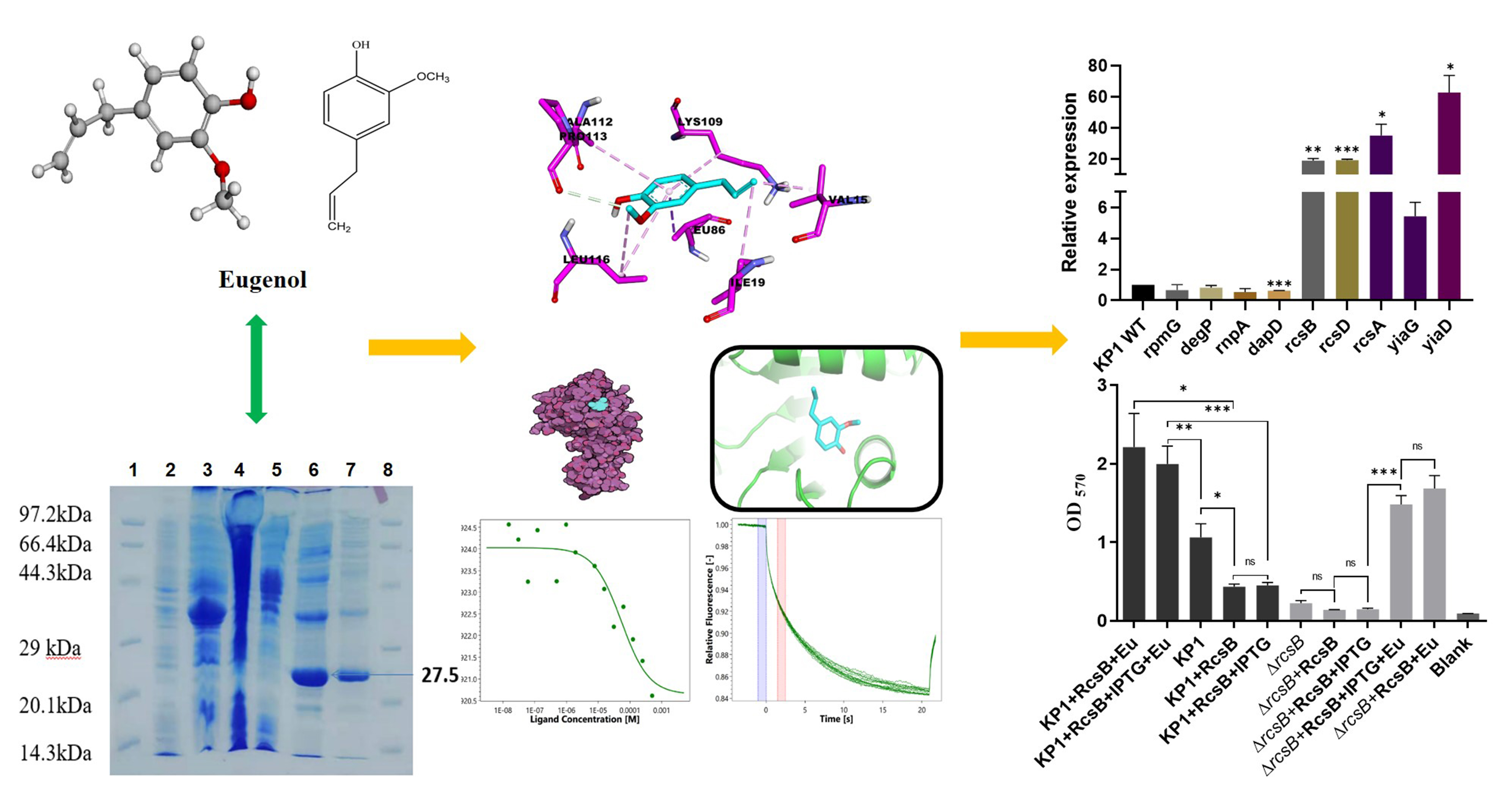

Supplement: Supplementary file 1 [file Image_1.JPG]
